# Optimized fertilization patterns increase foxtail millet biomass on the distribution and transformation in Loess Plateau of China

**DOI:** 10.1371/journal.pone.0318199

**Published:** 2025-02-07

**Authors:** Tianyou Zhou, Qinhui Liu, Shuangshuang Yang, Zhihao Wang, Panpan Zhang, Xiaolin Wang, Xiaoling Ji, Xiong Zhang

**Affiliations:** 1 College of Life Sciences, Yulin University, Yulin, China; 2 Engineering and Technology Research Center of Water Saving for Crops in Arid Area of Northern Shaanxi, Yulin, China; Mahishadal Raj College, Vidyasagar University, INDIA

## Abstract

Organic manure or microbial fertilizers are essential sources of plant nutrients to supplement farmland soil, and organic and inorganic fertilizers are considered an effective way to achieve sustainable agriculture by improving the soil and increasing crop yields. This experimental material was used foxtail millet (Setaria italica) “Changsheng 07”and started in the growing season of 2017–2018 in a dry farming area of northern Shaanxi Province, with five different fertilizing patterns, and each four repetitions, including T1(N,45kg·hm^-2^),T2(N,60kg·hm^-2^; P_2_O_5_ 30kg·hm^-2^),T3(N,90kg·hm^-2^;P_2_O_5_ 45kg·hm^-2^),T4(N,60kg·hm^-2^;P_2_O_5_ 40kg·hm^-2^,Organic matter 2000kg·hm^-2^),T5(N,60kg·hm^-2^;P_2_O_5_ 40kg·hm^-2^; microbial fertilizer 5kg·hm^-2^). The results showed that: (1) the above-ground dry matter accumulation with T4 and T5 increased by 15.04% and 33.68% during the flowering and pustulation stages, and the root/shoot ratio of T4 increased by 9.32% and 12.46% over two experimental years, respectively. (2) the leaf water use efficiency (WUE_L_) of T4 increased by 15.61%, 0.51% in two stages, respectively, (3) the yields and water use efficiency (WUE) of T3 exhibited a significantly increased by 11.06% and 37.61%, 9.50% and 37.51%, and increased stably by 9.23%-35.17% and 8.73%-35.11% in T4 and T5 respectively, over two seasons. In summary, applying organic and inorganic manure could effectively regulate the accumulation and distribution of above-ground biomass of foxtail millet, and ensure the high and stable grain yield.

## Introduction

Soil fertility is a comprehensive and objective reflection of the physical, chemical and biological properties of soil, and traditional agriculture apply chemical fertilizers to improve the comprehensive soil fertility condition and further increase crop yield [1, 2]. However, excessive synthetic fertilizer application brings more and more environmental and soil problems, leading to soil crusting, and provide nutrients for faster acidification, decline of both soil organic matter and fertility [3, 4], serious impact on agriculture and ecosystems environment [5, 6]. Thus, decreases in fertilizer usage and increase in fertilization efficiency are inevitable in the development of green agriculture [7].

Organic manure contains various bioactive substances and fungi that promote microbial reproduction and have multiple benefits in the agro-ecosystem, helps to activate soil nutrients rapidly, maintain soil fertility, contribute to the improvement of the root ecosystem, crop growth [8]. Agegnehu et al. reported that a long-term single used of chemical fertilizers causes a decline in soil health in terms of physical, chemical, and biological properties [9, 10]. Applying organic manure can improve crop growth by providing plant nutrients and improving the soil’s properties, thus providing a better environment for root development by improving soil structure [11]. However, signal application of inorganic fertilizers or organic manure can’t lead to sustainable productivity [12]. The combined application of organic and inorganic is a viable approach to overcome soil fertility limitations and improve agricultural crop productivity [13–15]. Therefore, the combined application of organic and inorganic fertilizers is essential to solve soil fertility depletion and sustainably increase crop productivity [16, 17].

The basis for forming dryland foxtail millet yield of dry matter accumulation, and nutrient organs such as roots, stems and leaves were important in the assimilation and transport of photosynthetic products [18]. The accumulation of photosynthetic substances is conducive to increasing the grain weight of winter wheat spikes at maturity [19]. It is closely related to above-ground biomass allocation with the level of crop yield [20]. Plants regulate the biomass allocation of various organs to maximize the use of light, nutrients and water resources. A reasonable fertilizer ratio plays a vital role in the growth of the foxtail millets’ above-ground part and enhances the yield [21]. Appropriate nitrogen and phosphate fertilizer can increase dry matter accumulation and WUE, and reduce soil water evaporation so that the total water consumption is reduced and the harvest index is improved to increase yield of foxtail millet [22, 23]. Alamzeb et al. demonstrated that combining organic and inorganic fertilizers improved dry matter allocation of wheat under a deep plowing system [24]. Applying different ratios of N, P, and organic manure can significantly affect maize growth, nutrient uptake, and dry matter yield [25]. Therefore, scientifically determining of fertilizer application and rational control of nutrient use efficiency are important ways to increase crop yield in various regions.

The current research on the growth and development of crops such as corn and wheat with the combination of organic and inorganic fertilizers is relatively comprehensive, but the systematic and correlated effects of the combination of inorganic fertilizers and microbial fertilizers on the growth and development process of dryland millet need to be further explored. Therefore, we used the foxtail millet cultivar was “Changsheng 07”. Under five different fertilizer ratios could promote the accumulation and distribution of the foxtail millet biomass, increase final yield and WUE in a dryland farm region in this paper. Under five different treatments, aiming at clarifying the differences of the combined application of organic and inorganic fertilizer modes on the biomass distribution of each organ of foxtail millet in dry land and the WUE, and choose a reasonable fertilizer distribution mode, to provide a theoretical basis for the high-yield and high-efficiency foxtail millet cultivation.

## Materials and methods

### Field experimental sites

**Fig 1:**
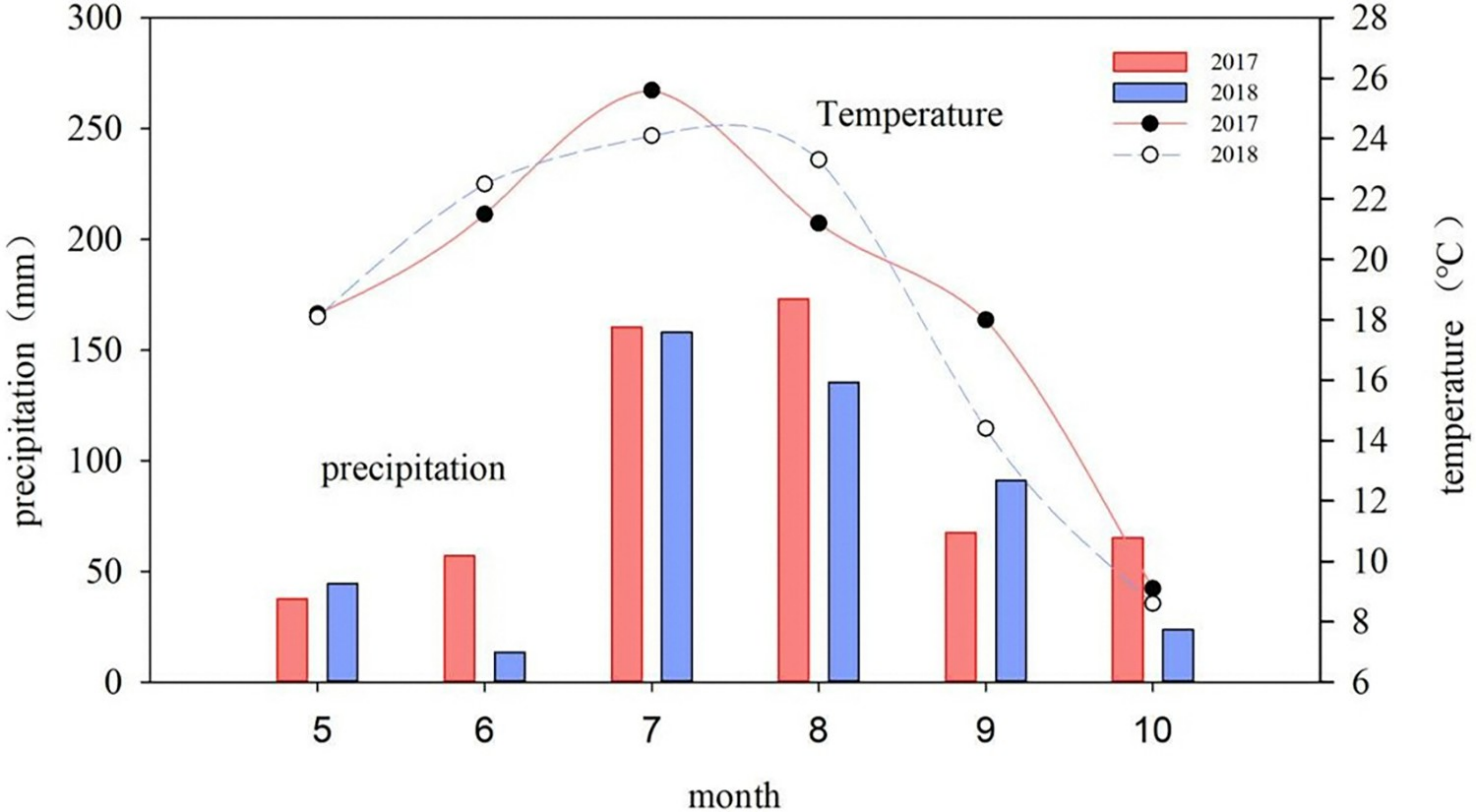
The precipitation and temperature during the foxtail millet in 2017–2018.

Field experiments took place in Shiyaoze Village of Hengshan District in Yulin City, Shaanxi Province Loess Plateau hilly and gully region in 2017 and 2018. The sowing time is from May 8–10th every year, and the harvesting time is from October 10–13th. The climate is temperate arid and semi-arid. The altitude is 1232 m, with four distinct seasons, solar radiation measurements, and an average annual temperature of 9.7°C (Fig 1). Frost-free period of about 146 d. More than 60% of the rainfall is concentrated in July-September. Annual evapotranspiration is 1211 mm. The soil is loessal soil. Organic matter content was 3.2 g kg^-1^, total nitrogen content was 0.3 g kg^-1^, alkaline dissolved nitrogen content was 18.9 mg kg^-1^, available phosphorus content was 6.2 mg kg^-1^ and potassium content was 66.0 mg kg^-1^.

### Materials

The foxtail millet cultivar “Changsheng 07” selected for the experiment is a new variety of high-quality, high-yield, multi-resistant by Millet Research Institute, Shanxi Academy of Agricultural Sciences. The seed was mutated by Changnong 35 variant strain through selection and breeding. This variety has a growth period of about 125 days, with excellent comprehensive characteristics, strong adaptability, and yield stability.

Nitrogen fertilizer was produced by the Inner Mongolia Boyuan International Agricultural Co., Ltd., Ordos City, China (urea N ≥ 46.6%). Phosphate fertilizer was produced by Hanzhong Tangfeng Chemical Co., Ltd. Hanzhong, China (P_2_O_5_ ≥ 12.0%), organic matter (organic matter ≥ 30%, NPK ≥ 3%), The microbial manure was produced by Shandong Lvlong Biotechnology Co., Ltd. (the content of effective number of live bacteria ≥ 20 billion·g^-1^).

#### Experimental design

This field experiment was located at the intersection of the Loess Plateau and the Mu Us Sandy Land, conducted in a mulched randomized block design, adopting five fertilization models (Table 1). The soil content organic matter was lower (nearly 0.3%), the soil content availability nutrients was lower, the mircroal function was weak. The experiment used organic manure, mircobial manure substitute for partial replacement of chemical fertilizer, though added organic matter, mircobial diversity to activating soil intrinsic nutrient availability. Achieving the combination of land use and maintenance. Finally, the simultaneous improvement of organic matter content, microbial diversity, and crop yield.

**Table 1:** The experimental design of fertilizer combined models (kg·ha^-2^).

Treatment	N	P_2_O_5_	Organic manure	Microbial manure
T1	45	0	0	0
T2	60	30	0	0
T3	90	45	0	0
T4	60	40	2000	0
T5	60	40	0	5

The plot area was 20 m^2^, 4 m× 5 m, each and treatments is repeated 4 times. The distance between each plot was 0.5 m, plant spacing of 0.25 m. The foxtail millet inter-membrane ridging is 0.4 m wide at the bottom of the ridge, 0.08–0.10 m wide at the height of the ridge. Bunch planting on the 0.03–0.05 m film side. After 4–6 leaves of seedlings emerge, appropriate thinning and seeding should be carried out according to the planting density (180000 plants·ha^−1^).

### Determination indexes and method

#### Electrical conductivity

Measure the electrical conductivity of foxtail millet during the flowering and pustulation stages. Weigh 4 g of air-dry soil and place it in triangle bottle. Add 20 ml of water (water to soil ratio of 5:1), plug it with a stopper, shake for 3 minutes, settle and clarify, and measure directly without filtering. Measure the temperature of the liquid. Rinse the electrode with the test solution 1–2 times, dry it with filter paper, and then insert the electrode into the test solution until the platinum plate is completely submerged under the liquid surface. Repeat each sample 2–3 times.

#### Measurement of above-ground dry matter

During the two-year experiment, five representative plants were collected from each plot at the flowering and pustulation stages of foxtail millet. The different organs (stems, leaves and spike) of each plant were separated, put into the oven in batches, deoxidized at 105°C for 30 min, and the samples were baked until constant weight at 75°C, and then obtain the biomass of stems, leaves and spikes.

#### Determination of root dry weight

In the pustulation stage, the root drill method was used to obtain all the roots in the 0–30 cm plow layer. We used a nylon mesh sieve to remove most of the soil and then washed the samples under a slow flow of water, removing the residual soil with a brush. The zip lock bag was stored at 4°C in a fridge. The root was put into thickened zip lock bags at 75°C until constant weight, and then weighed to the root biomass.

#### Determination of chlorophyll and photosynthetic indexes

During the flowering and pustulation stages of foxtail millet, the chlorophyll relative content of the apical fully expanded leaves was determined by using SPAD 502 plus (Konica Minolta, Japan). 15 foxtail millet were selected for each treatment, and 5 mature leaves were selected for each foxtail millet. Each leaf was determined 3 times to obtain the average value. Leaf photosynthetic indexes were measured using Li-6400 photosynthesize (Li-COR, USA), 5 representative plants were selected for each plot, and the photosynthetic rate and transpiration rate were measured on the top mature leaves to calculate the instantaneous WUE_L_.

#### Determination of yield and yield components

Twenty samples-plant samples were randomly collected from the middle of each plot after foxtail millet at maturity. The sample spikes were naturally dried, its weight per spike (g) and 1000-millet weights (g) of the sampled foxtail millet (20 plants) in each plot were weighed with an electronic balance, calculated the total yield of the subplot and conversion to yield per unit area.

#### WUE calculation

Soil water content from 0–100 cm was measured at the early sowing and maturity stages, with each sampling level being 10 cm, collected and placed in an aluminum box. After collected and placed in an aluminum box. Then soil water content was calculated after drying in the laboratory. The soil water content (W),

$$W=\frac{W_{1}-W_{2}}{W_{2}-W_{0}}\times 100\%$$ (1)

W: soil moisture content (%), W_0_ is aluminum box weight (g), W_1_ is wet soil weight (g), W_2_ is dry soil weight (g)

ET: evapotranspiration (mm):

$$ET=\Delta W+P\;\Delta W=W_{1}-W_{2}$$ (2)

P: the effective precipitation during the growth period of foxtail millet, ΔW: change in soil water storage (mm), W_1_: soil water storage early sowing period, W_2_: soil water storage harvest period.

WUE = water use efficiency (kg·mm^−2^ ha^−1^)

$$WUE=\frac{Y(kg\cdot ha^{-1})}{ET(mm)}$$ (3)

ET = evapotranspiration (mm); Y = yield (kg·ha^−1^).

#### Data analysis and statistics

SPSS 27.0 for one-way ANOVA and correlation analysis; The figures using Origin 2017 and SigmaPlot 12.5.

## Results

### Effect of different fertilizer patterns on soil moisture content, temperature and electrical conductivity of foxtail millet

**Fig 2:**
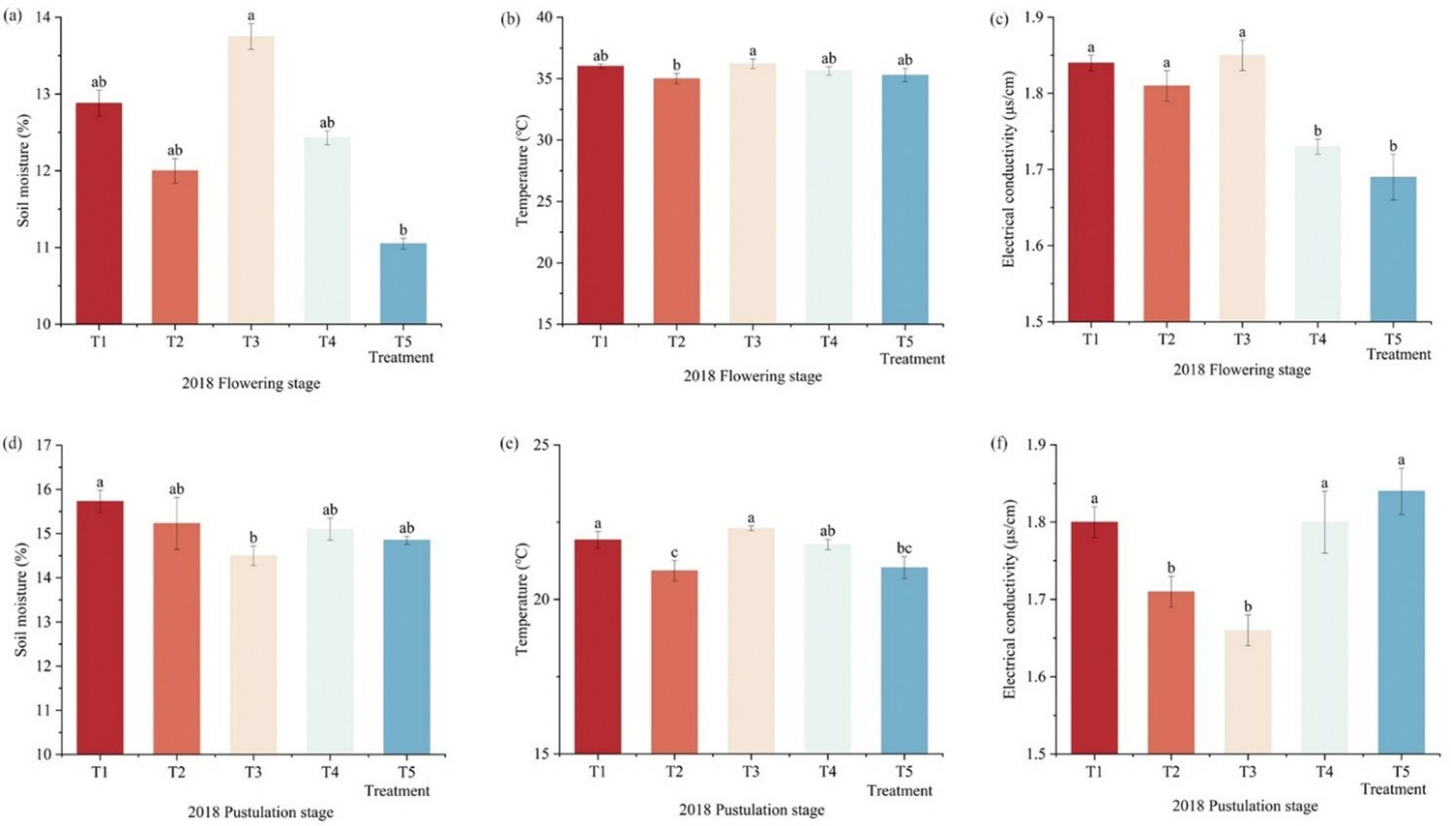
The effect of fertilizer application patterns on soil moisture, temperature, electrical conductivity of foxtail millet. Note: (a) is soil moisture during the flowering stage in 2018, (b) is soil temperature during the flowering stage in 2018, (c) is soil electrical conductivity during the flowering stage in 2018, (d) is soil moisture during the pustulation stage in 2018, (e) is soil temperature during the pustulation stage in 2018, (f) is soil electrical conductivity during the pustulation stage in 2018.

Compared with T1, the soil moisture content was decreased by 7.33%, 3.62%, 14.21%, 3.28%, 4.17%, and 5.93% at T2, T4, and T5 in the flowering and pustulation stages, respectively (Fig 2). At the flowering stage, T3 soil water content reached a maximum of 13.75%. Whereas compared with T1, T3 was significantly decreased by 8.48% in the pustulation stage. Under different fertilizer application patterns, which showed a decreasing trend, then increasing and then decreasing on the soil temperature. In the flowering stage, there was no significant different from treatments. Compared with T1, T2 and T5 were significantly decreased by 4.78% and 4.10%, respectively, in the pustulation stage. The electrical conductivity significantly decreased by 6.36% and 8.15% in T4 and T5 compared with T1 in the flowering stage. As the fertility progressed to the foxtail millet pustulation stage, the electrical conductivity increased by 2.17% in the T5, with no significant difference from T4, and T3 significantly decreased by 8.43%, compared with T1 (p<0.05).

### Effects of different fertilization patterns on chlorophyll relative content of foxtail millet

**Fig 3:**
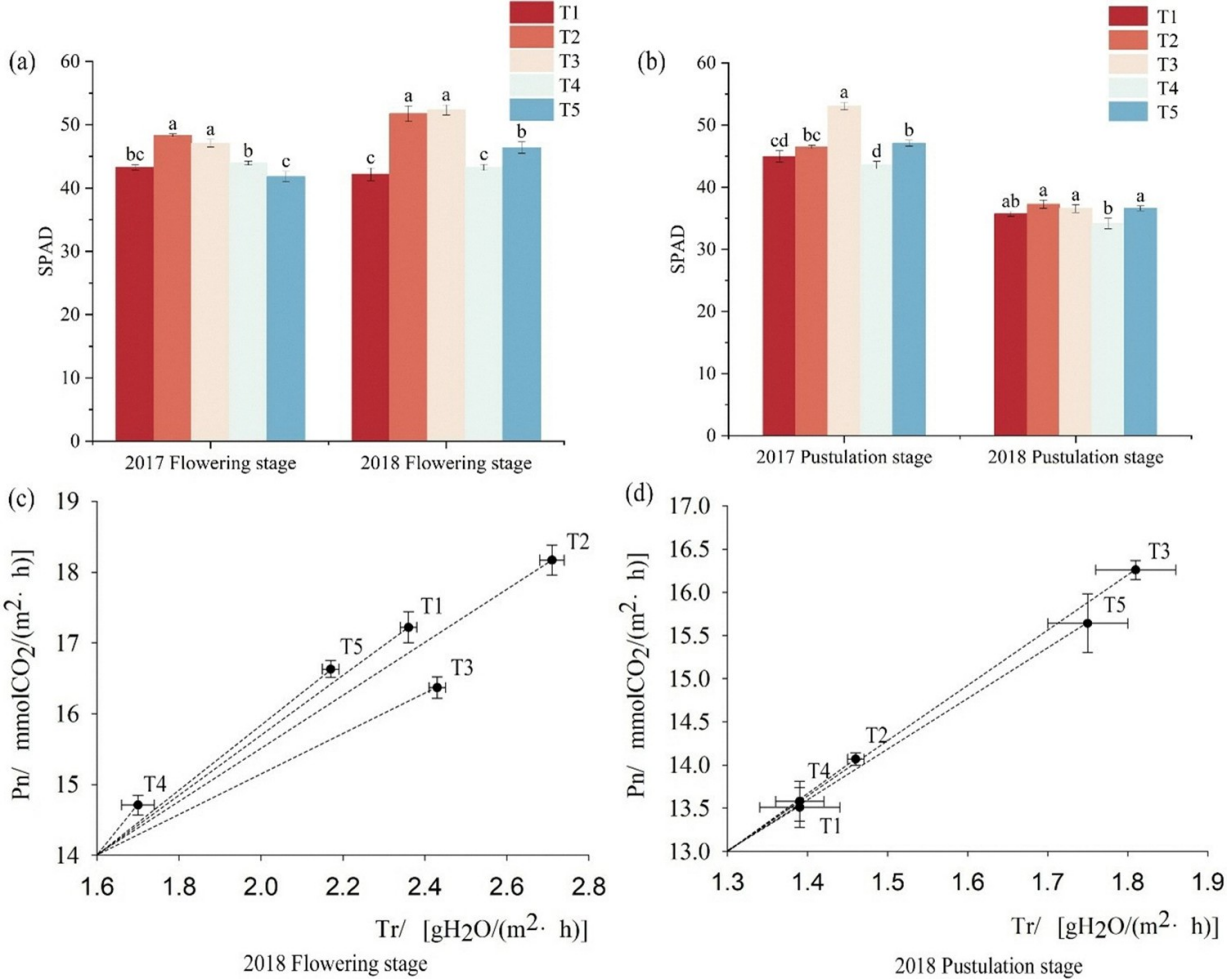
The effects of fertilizer models on chlorophyll relative content of foxtail millet. Note: Different lowercase letters in the upper part of the bar chart indicated significant difference at ɑ = 0.05. Bar indicate SE (the same below). (a) is SPAD during the flowering stage of 2017–2018, (b) is SPAD during the pustulation stage of 2017–2018, (c) is WUE_L_ during the flowering stage in 2018, (d) is WUE_L_ during the pustulation stage in 2018.

Optimization of fertilization strategies can effectively improve leaf chlorophyll relative content, thereby enhancing leaf photosynthetic physiological function and WUE_L_, during the flowering stage in foxtail millet (Fig 3). The SPAD for T3 was significantly higher than the other four treatments. During the flowering stage, compared with T1, the SPAD for T2 and T3 increased by 8.07%-19.40% (P < 0.05). There was no significant difference between the T4 and T1, which increased by 1.52% and 2.47%, respectively. At the flowering stage, compared with T1 the SPAD for T5 decreased by 3.54% in 2017 and significantly increased by 9.06% in 2018. At the pustulation stage, compared with T1, T3 significantly increased by 15.31% and 2.33%, while that of T4 was not significantly, decreased by 3.17% and 4.54%, and the SPAD for T5 increased by 4.57% and 2.46%, respectively.

At the flowering stage, compared with inorganic fertilizers, the photosynthetic and transpiration rates for the combined organic and inorganic fertilizers application were decreased. The WUE_L_ for chemical fertilizer with organic or microbial manure increased by 4.70–15.61%. During the pustulation stage, the photosynthetic and transpiration rates for T3 reached the maximum. The photosynthetic and transpiration rates of T4、T5 increased by 20.57%, 0.52% and 13.62% compared with control, and the WUE_L_ of chemical fertilizer with organic manure increased by 0.51%.

### Effect of five fertilizer models on above-ground biomass of foxtail millet

**Fig 4:**
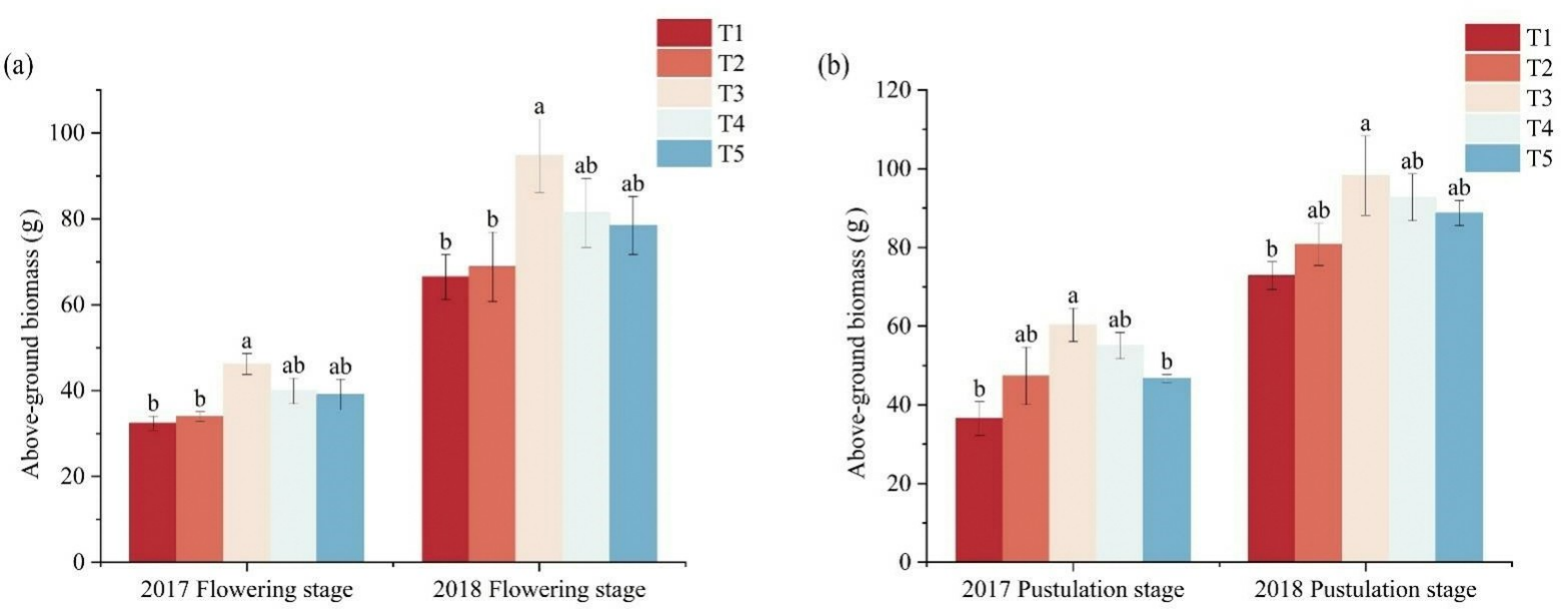
The effects of fertilizer models on aboveground biomass of foxtail millet. Note: (a) is the accumulation of above-ground biomass during the flowering stage of 2017–2018, (b) is the accumulation of above-ground biomass during the pustulation stage of 2017–2018.

Fertility affects the accumulation of dry matter and the growth of foxtail millet (Fig 4). With different fertilization modes, the above-ground biomass showed a trend of decreasing, then increasing and then decreasing, and the above-ground biomass for the T3 reached the maximum, during the flowering and pustulation stage. Compared with the T1 and T2, the above-ground biomass for the T3 significantly increased by 29.84%, 26.59%, 29.86%, and 27.34% (p < 0.05), and compared to T1, T4 and T5 increased by 15.04%-18.80%, during the flowering stage. Compared with T1, T3 was significantly increased by 39.47%, 25.87%, respectively (P < 0.05), during the two years of pustulation stage. There was no significant difference between T4 and T5 and T1, which increased by 17.92%-33.68%.

### Effects of different fertilization patterns on the proportion of biomass allocation in the aboveground foxtail millet

**Fig 5:**
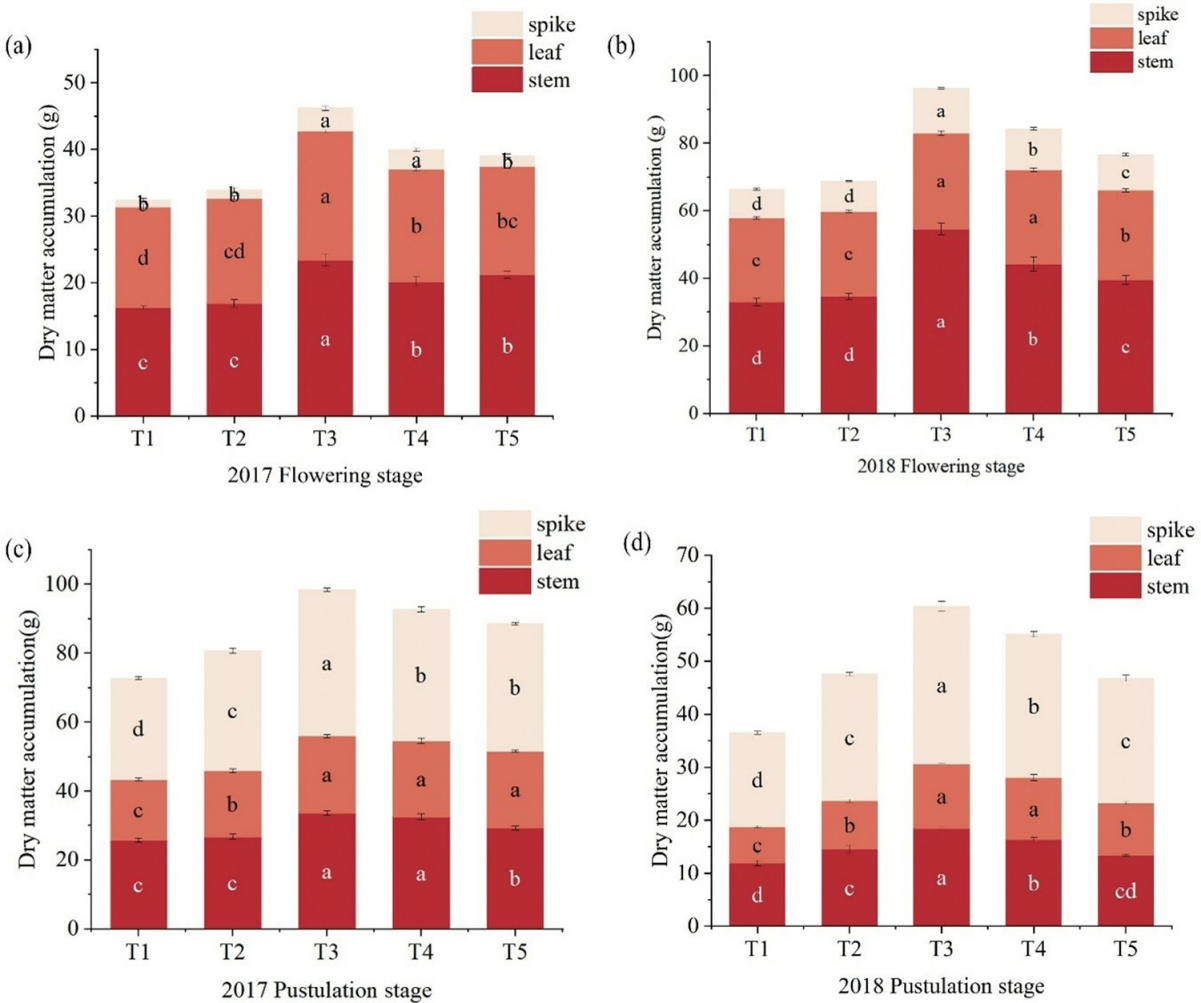
The effect of five fertilizers on dry matter accumulation. Note: (a) is the dry matter accumulation during flowering stage in 2017, (b) is the dry matter accumulation during flowering stage in 2018, (c) is the dry matter accumulation during pustulation stage in 2017, (d) is the dry matter accumulation during pustulation stage in 2018.

As can be seen from Fig 5, with the advancement of the fertility period, the changes in dry matter allocation of above-ground organs of foxtail millet showed a consistent pattern between the two years. The above-ground dry matter accumulation of T3 reached the highest, in the flowering and pustulation stages over two years. Compared with T1, the average values of stem, leaf and spike dry matter accumulation of T4 and T5 were significantly increased by 19.13%, 10.81%, 63.86%, 23.14%, 7.25%, 34.81%, respectively (p<0.05). At the same time, that of T2 was no significantly different from T1, in the flowering stage of 2017–2018. The above-ground dry matter accumulation was gradually transformed into a spike during the pustulation stage, compared with T1, the stem, leaf, and spike of T4 and T5 were significantly increased by 11.12%-27.11%, 20%-41.45%, and 20.62%-35.21%.

### The effects of fertilizer models on the foxtail millet root/shoot ratio

**Fig 6:**
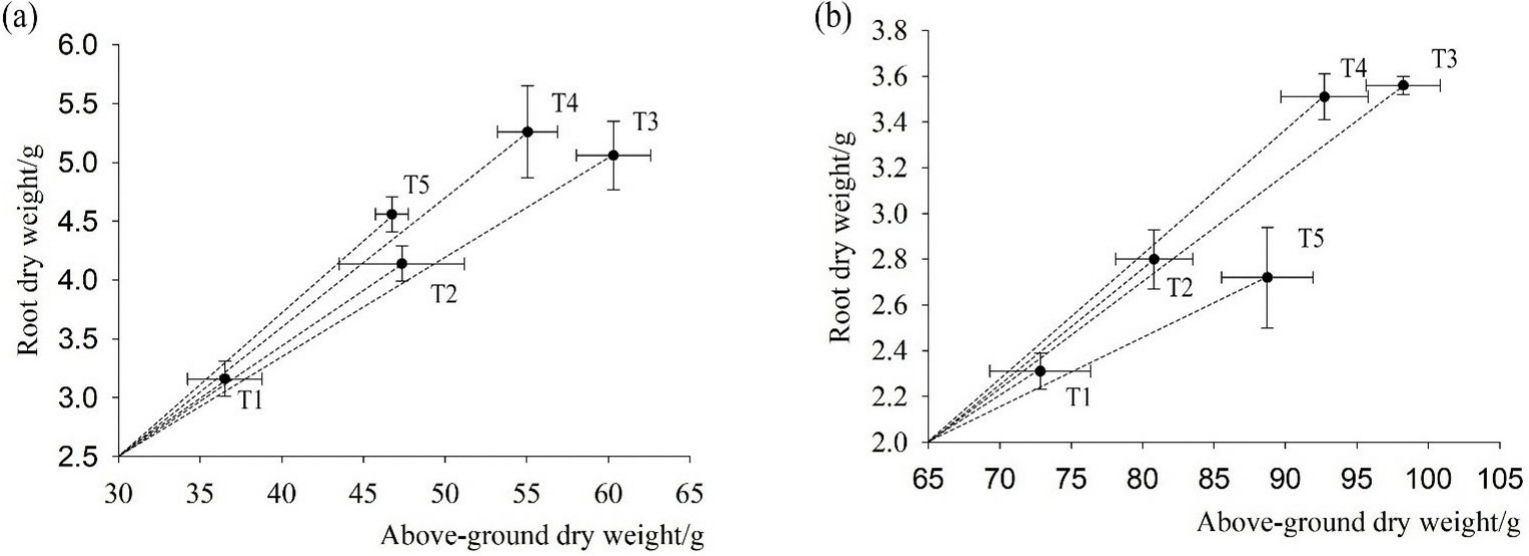
The effects of five fertilizers models on root-crown of foxtail millet. Note: (a) is root/shoot ratio during the pustulation stage of 2017, (b) is root/shoot ratio during the pustulation stage of 2018.

Chemical fertilizers with organic or microbial manure could significantly increase the root/shoot ratio of foxtail millet in the pustulation stage of foxtail millet (Fig 6). There was sufficient rainfall in 2017, compared to T1, T4 and T5 were increased by 9.32% and 11.27%, in root dry weight was generally high, and nitrogen and phosphorus fertilizers with organic or microbial manure had a high root/shoot ratio. There was less rainfall in 2018, and the root/shoot ratio for T2, T3, and T4 increased by 8.47%, 12.46% and 16.21%, respectively, and T5 decreased by 3.47%. Chemical fertilizer with organic manure significantly increased root dry weight by 34.19% and microbial manure by 15.07%. Fertilization strategy adapted to seasonal drought by regulating the root system.

### Correlation between dry matter and yield under different fertilizer application

#### Correlation between stem dry weight and yield

**Fig 7:**
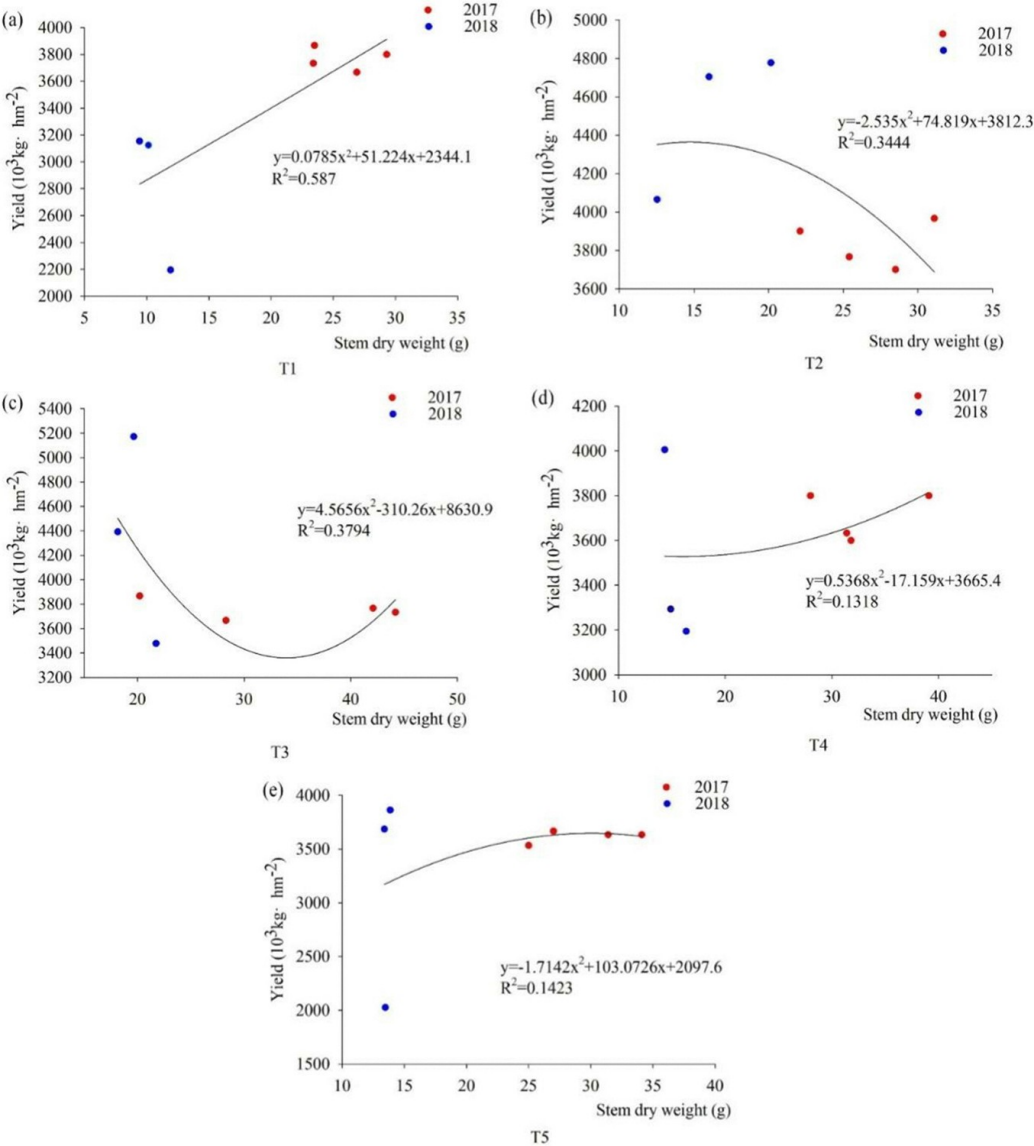
Fitting curves of the correlation between stem dry weight and yield under different fertilization patterns. Note: (a) is the correlation between T1 stem dry weight and yield during the pustulation of 2017 to 2018, (b) is the correlation between T2 stem dry weight and yield during the pustulation of 2017 to 2018, (c) is the correlation between T3 stem dry weight and yield during the pustulation of 2017 to 2018, (d) is the correlation between T4 stem dry weight and yield during the pustulation of 2017 to 2018, (e) is the correlation between T5 stem dry weight and yield during the pustulation of 2017 to 2018.

In the pustulation stage, the correlation curve between stem dry weight and yield showed different trends under different fertilization patterns during 2017 to 2018 (Fig 7). With the foxtail millets’ stem dry weight were increased, the yield increased between T1 and T4, in which T4 increased more slowly. T2 showed a decreasing trend, T3 showed a decreasing and then an increasing trend, and it reached the lowest in the middle of the stem dry weight of 30-40g. T5 showed increasing and then decreasing, and when stem dry weight was 27g, stem dry weight and yield were decreased. Under five different fertilization patterns, the nitrogen fertilizer R^**2**^ maximum value between stem dry weight and yield when is 0.59.

#### Correlation between leaf dry weight and yield

**Fig 8:**
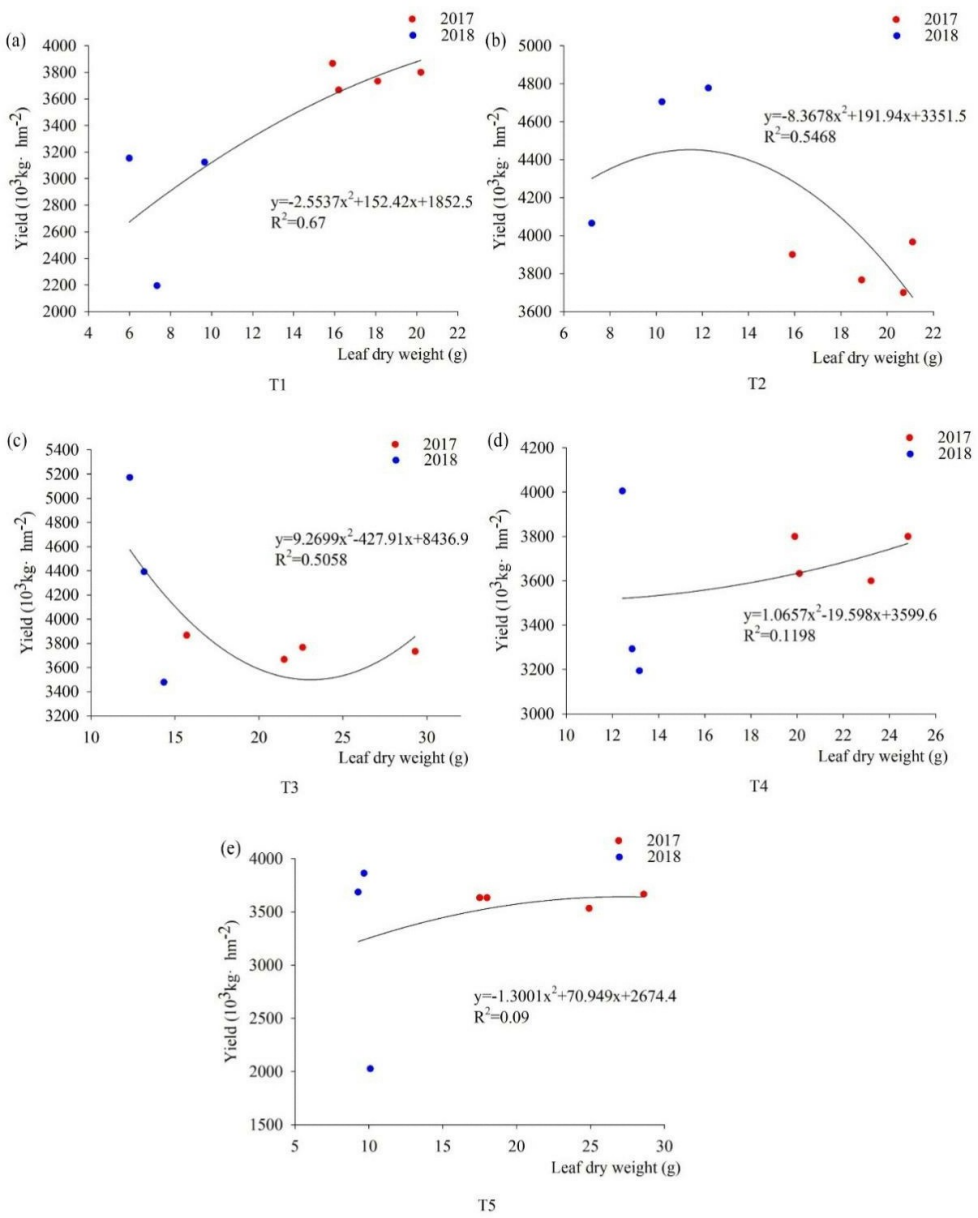
Fitting curves of the correlation between leaf dry weight and yield under different fertilization patterns. Note: (a) is the correlation between T1 leaf dry weight and yield during the pustulation of 2017 to 2018, (b) is the correlation between T2 leaf dry weight and yield during the pustulation of 2017 to 2018, (c) is the correlation between T3 leaf dry weight and yield during the pustulation of 2017 to 2018, (d) is the correlation between T4 leaf dry weight and yield during the pustulation of 2017 to 2018, (e) is the correlation between T5 leaf dry weight and yield during the pustulation of 2017 to 2018.

The correlation curves of leaf dry weight and yield and stem dry weight and yield showed the same trend under different fertilization patterns, during the foxtail millet from 2017 to 2018 (Fig 8). T1 showed an increasing trend in leaf dry weight and yield. When the leaf dry weight was 20g, the yield increased slowly and showed a decreasing or stabilizing trend in T1, and compared with the other four treatments, R^**2**^ reached a maximum of 0.67. With the increase of leaf dry weight, the yield of T2 shows a trend of first increased and then declined. The correlation curve with leaf dry weight and yield of T3 showed a trend of decreasing and then increasing. T4 leaf dry weight increased and yield increased, and T5 showed a trend of increasing and then with the increase of leaf dry weight yield remained stable.

#### Correlation between root dry weight and yield

**Fig 9:**
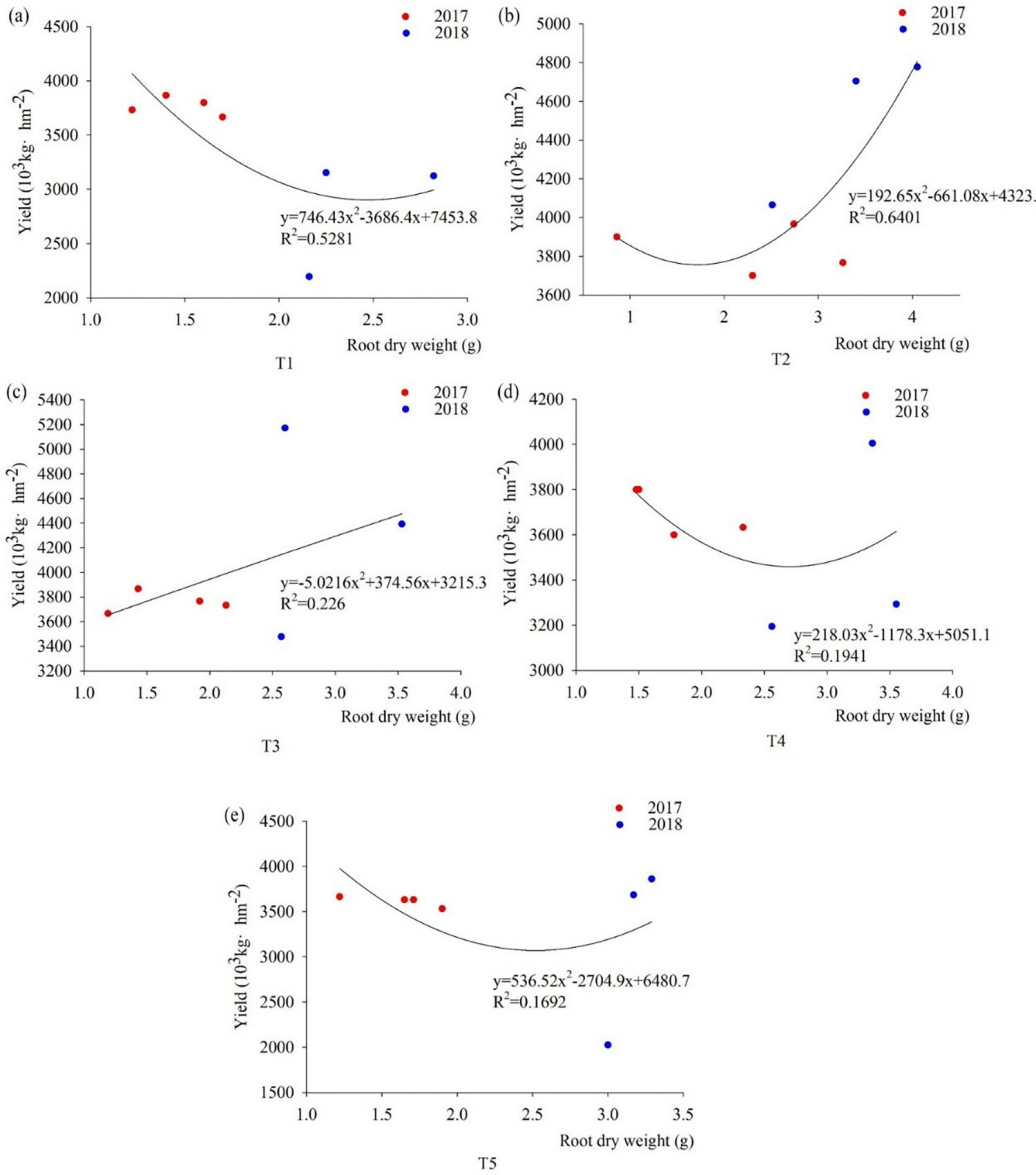
Fitting curves of the correlation between root dry weight and yield under different fertilization patterns. Note: (a) is the correlation between T1 root dry weight and yield during the pustulation of 2017 to 2018, (b) is the correlation between T2 root dry weight and yield during the pustulation of 2017 to 2018, (c) is the correlation between T3 root dry weight and yield during the pustulation of 2017 to 2018, (d) is the correlation between T4 root dry weight and yield during the pustulation of 2017 to 2018, (e) is the correlation between T5 root dry weight and yield during the pustulation of 2017 to 2018.

The four treatments T1, T2, T4, and T5 showed a decreasing and then increasing trend in root dry weight and yield, among them, R^2^ of T2 being the largest at 0.6401 compared to the other four treatments (Fig 9). During the pustulation, the foxtail millet roots dry weight was increases, the yield of T3 increases from 2017 to 2018.

#### The effects of fertilizer models on foxtail millet yield components

In 2017–2018, compared with T1, the spike length of T4 and T5 increased by 6.77%, 6.22%, 5.85%, and 4.83%, respectively (Table 2). With the change in fertilizer application patterns, spike diameters showed increasing and decreasing trends. Compared with T1, the spike diameters were no significant different from that of T4 and T5 increased by 11.06%-15.38%, and T3 increased significantly by 17.72%, in 2018. From 2017 to 2018, 1000-grain weight of T4、T5 were only increased by 0.90%-2.25%, compared with nitrogen fertilizer. The grain weight per spike of T3 was significantly increased by 37.47%-43.92% and T4 and T5 increased by 19.25%-35.03%. In 2017–2018, there was a significant effect (P < 0.01) on 1000-grain weight and grain weight per spike. There was a significant effect (P < 0.01) on spike length, spike diameter, and weight per spike between treatments, but there was no significant effect on 1000-grain weight (P>0.05). The interaction of different treatments between the two years only significantly affected grain weight per spike, and there was no significant effect on spike length, spike diameters and 1000-grain weight (P>0.05).

**Table 2:** The effects of fertilizer models on yield composition.

Year	Treatment	Panicle Length (cm)	Panicle Diameter (mm)	1000-Grain Weight (g)	Grain Weight per panicle (g)
2017	T1	17.34±0.34c	2.14±0.08a	3.30±0.10ab	12.73±0.58c
	T2	18.14±0.47bc	2.18±0.02a	3.37±0.07ab	16.03±0.64bc
	T3	19.44±0.14a	2.30±0.04a	3.53±0.07a	22.70±1.79a
	T4	18.60±0.45ab	2.25±0.01a	3.27±0.07b	16.80±0.61bc
	T5	18.49±0.05ab	2.22±0.11a	3.33±0.03ab	18.80±2.17ab
2018	T1	16.74±0.03c	2.09±0.11b	3.04±0.04a	15.69±1.75b
	T2	20.68±0.22a	2.21±0.18ab	3.11±0.09a	17.73±3.25ab
	T3	20.11±1.46ab	2.54±0.08a	3.22±0.08a	25.09±1.26a
	T4	17.78±1.09bc	2.47±0.13ab	3.08±0.06a	24.15±2.72a
	T5	17.59±0.62bc	2.35±0.07ab	3.11±0.11a	19.43±1.42ab
Variation source					
Year		NS	NS	**	*
Treatment		*	*	NS	**
Year ×Treatment		NS	NS	NS	*

Note: Means of replicates ± standard error, the different letter indicate the significant differences (P<0.05). *: Significant difference at the 0.05 level; **: Significant difference at the 0.01 level; NS: No significant difference (the same below).

#### Effect of different fertilizer models on WUE and yield of foxtail millet

According to the differences between year and rainfall, the water consumption of foxtail millet in the two years (formula 1–2). The yield composition can effectively illustrate that optimizing the fertilizer application patterns can increase grain yield. As seen in Table 3, compared with nitrogen fertilizer, the combined application of nitrogen and phosphorus fertilizer has achieved the highest yield for two years, with a significant increase by 11.06% and 37.61%. T4、T5 were significantly increased by 9.23% -35.17% of crop yield. With the change in fertilizer application patterns, WUE showed a trend of first increasing and then decreasing in both years (formula 3), the nitrogen phosphorus fertilizer combined with organic manure or fertilizer were increased by 8.73% -35.11%. In 2017, the WUE for chemical fertilizer with organic manure reached a maximum value of 7.11 kg·mm^**−2**^ ha^**−1**^.

**Table 3:** Effects of organic and inorganic fertilizer on foxtail millet WUE and yield.

Treatment	Water consumption/(mm)		Yield/(×10^3^ kg·ha^-2^)		WUE/(kg·mm^−2^ ha^−1^)	
	2017	2018	2017	2018	2017	2018
T1	554.82	491.76	3.54±0.11b	2.82±0.17c	6.38±0.20b	5.73±0.03c
T2	564.12	492.04	3.77±0.13b	3.50±0.03b	6.68±0.23b	7.11±0.06b
T3	564.21	493.06	3.98±0.06a	4.52±0.03a	7.05±0.11a	9.17±0.06a
T4	558.45	492.88	3.97±0.10a	4.35±0.04a	7.11±0.18a	8.83±0.08a
T5	557.99	492.37	3.90±0.10a	4.19±0.15a	6.99±0.18a	8.51±0.30a

#### The correlation between yield components

**Fig 10:**
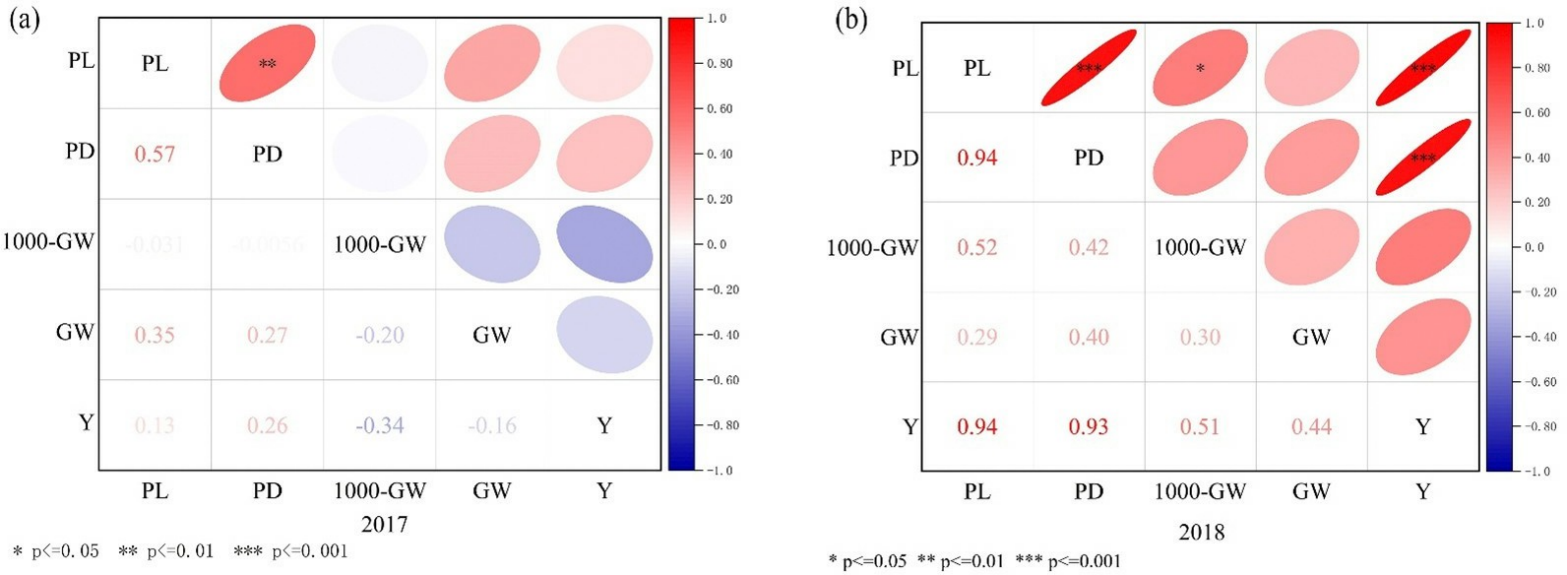
Correlation analysis between yield components. Note: *** significant difference at p<0.001; ** significant difference at p<0.01; * significant difference at p<0.05. PL is Panicle Length, PD is Panicle Diameter, 1000-GW is 1000-Grain Weight, GW is Grain Weight per panicle, Y is yield. (a) is the correlation between yield components in 2017, (b) is the correlation between yield components in 2018.

There was a clear relation between yield components during 2017–2018 (Fig 10). In 2017, PL and PD were highly significant correlations (p < 0.01). PL, PD and GW, PL, PD and Y were a positive correlation, while the rest are negatively correlated. In 2018, there were positive correlations between the PL and PD (0.94), the PL and Y (0.94), the PD and Y (0.93) (p < 0.001). The PL and 1000-GW (0.52) were significant correlations (p < 0.05). The rest indexes were a negatively correlation.

## Discussion

The chlorophyll relative content of leaves is an important indicator, its will directly affect the plant’s absorption, utilization, and conversion of delayed leaf senescence, and reflecting the physiological condition of the crop and the functional material basis for photosynthesis [26, 27]. In general, the higher net photosynthetic rate and transpiration rate of a crop indicate that it promotes respiration, improves the efficiency of the plant’s utilization of light energy, increases dry matter accumulation, and makes it easier to obtain high yields [28, 29]. Nitrogen fertilization may improve the ability of crops to produce chlorophyll, which is one of the most significant active factors regulating the photosynthetic capacity of crop leaves [30, 31]. The moderate application of nitrogen fertilizer is a feasible way to increase the rate of aboveground photosynthesis in crops, improve dry matter development and translocation and promote plant growth and yield [32]. Kubar et al. researched that the photosynthetic rate usually increased and stabilized; the intercellular CO_2_ concentration, transpiration, and net photosynthetic rate usually increased and then decreased after the application of nitrogen fertilizer, the light transfer efficiency of high nitrogen levels for winter wheat production is significantly better than that of low nitrogen levels, indicating that sufficient nitrogen fertilizers can significantly enhance the net photosynthetic characteristics in the flag leaf of winter wheat [33]. Compared with the full application of inorganic nitrogen fertilizer treatment, organic manure and inorganic fertilizer can prolong the duration of green leaves in maize, delay the aging and shedding of maize leaves, and maintain a larger photosynthetic area in the later stage of maize growth [34, 35]. It can significantly improve the SPAD, Pn, Gs, and Tr of maize throughout the growth period, reduce the Ci of maize, enhance the photosynthesis of maize leaves, and facilitate the distribution of photosynthetic products to grains in the later stage [36]. The combined application of organic and inorganic fertilizers increased photosynthetic rate and stomatal conductance, with a high correlation coefficient between yield and photosynthetic rate (r = 0.926, p < 0.001), and increased maize yield [37]. The results of this study showed that SPAD, with the advancement of the fertility period, showed a decreasing trend. Compared with nitrogen fertilizer, the chemical fertilizer with organic manure increased the SPAD, and the WUE_L_ reached the maximum. Combining organic and inorganic fertilizers can maintain higher photosynthetic capacity during the pustulation stage and further enhance yield (Fig 3).

Stems and leaves are important nutrient organs of plants, Stems serve as crucial pathways for transporting water and nutrients in plants, while leaves are vital organs for absorbing light energy and synthesizing carbohydrates [38]. The relative biomass of stems and leaves reflects the morphological adaptations of plants to their environment through self-regulation [39, 40]. At all stages of plant development, the application of organic-inorganic fertilizers increases the content of soil organic matter, enhances soil fertility, facilitates balanced nutrient absorption by crops, and promotes dry matter accumulation in plant bodies [41, 42]. Yang et al. showed that the combined application of organic and inorganic fertilizers resulted in lower ammonia volatilization losses than urea, leading to higher ammonium and nitrate nitrogen content in the soil, increased nitrogen uptake, dry matter accumulation in maize, and higher yields [43]. Under the same nitrogen application rate, replacing some chemical fertilizers with organic fertilizers significantly increases the dry matter accumulation of spring maize [44]. Organic manure and 50% organic fertilizer instead of chemical fertilizers significantly improved the proportion of dry matter in the upper part of rice stems, leaves, and panicles, coordinated the proportion of dry matter in various organs, and increased the proportion of dry weight in panicles, which is beneficial for increasing rice yield [45]. In this study, compared with T1, the aboveground biomass of T3 (N: P is 90:45) significantly increased by 25.87%- 39.47%, during the flowering and pustulation stages. The combination of organic and inorganic fertilizers can effectively regulate the accumulation and distribution of dry matter in the crop so that it can be directly transferred to the spikes and then increased in crop yield (Figs 4 and 5). Reasonably, the application of fertilizer patterns can significantly increase soil organic matter content, improve the soil environment, promote the growth and development of the root system, and increase crop yields [46]. Zhou et al. also found that combining chemical fertilizer with manure promoted root growth and development, improve root vitality of foxtail millet [47]. Liao et al. showed that 25% inorganic fertilizer combined with 75% organic fertilizer (C0.25O0.75) effectively increased in 0–1 mm fine roots, and improved the proportion of absorbed roots, increased root dry weight [48].Weng et al. research on rice showed that the appropriate combination of organic-inorganic fertilizers can greatly enhanced the root activity of rice, improved dry matter accumulation and distribution patterns, thereby increased the yield of main and regenerated rice crops [49]. In this study, the combined organic and inorganic fertilizer promotes the root system dry matter, with an increase of 30.70%-39.92%, and a significant increase in the root-shoot ratio in 2017 (Fig 6). With the advancement of the fertility stage, the accumulation of dry matter in the above-ground part of the crop with the combined application of organic and inorganic fertilizers is stabilized. Due to the role of organic and inorganic fertilizers in soil improvement, foxtail millets’ above-ground dry matter accumulation increased to adapt to drought, providing sufficient carbohydrates for yield improvement in 2018 (Figs 4 and 5).

Soil moisture is the most critical factor affecting crop growth and development, and using limited natural precipitation is of great significance for increasing production and efficiency in agriculture in semi-arid areas [50, 51]. Organic fertilizers tend to the C and N cycles, resulting in soil N mineralization to immobilization turnover [52]. Combining nitrogen fertilizers with organic manure also affects the decomposition rate of organic manure by regulating the soil carbon-to-nitrogen ratio, thereby altering the nutrient supply and consequently affecting soil water uptake and crop yields [53]. Compared with the application of inorganic fertilizers, the combination of organic and inorganic fertilizers has higher soil water storage capacity and soil moisture content, which can efficiently utilize precipitation and fertilizer resources [54]. Zhai et al. showed that organic manure replacing some chemical fertilizers’ soil moisture content, yield, and WUE were all improved; 30% of organic manure replacing chemical fertilizers had a more significant potential to reduce the nitrogen fertilizer applied [32]. Compared to the control, the soil conductivity and soil moisture content were increased with 100% NPK+ organic fertilizer and enhanced crop roots to improve soil physical conditions during 28 years soybean wheat maize rotation [55]. And depth of soil layer from 0 to 10 cm, organic fertilizer (1.56 d S m-1) has an EC 2.2 times higher than inorganic fertilizer (0.71 d S m-1) [56]. In my experimental indicated that long-term application of nitrogen fertilizer, WUE has been improved and the average increased by 4.49% -37.51% for both years. The water use efficiency of the combination of organic and inorganic fertilizers is lower than that of inorganic fertilizers, this reason may increase soil water retention capacity (Table 3).

The application of organic fertilizer can maintain or increase grain yield, mainly because it can improve soil fertility, promote nutrient availability [57]. Yang et al. found that the combination of organic and inorganic fertilizers has a positive effect on the weight, quantity, size, element content, and total yield of tomato fruits [58]. Replacing an appropriate amount of inorganic nitrogen fertilizer with organic fertilizer can increase crop yield by 3.3% [59]. Replacing some inorganic nitrogen fertilizer with organic fertilizer ensures and even increases winter wheat yield, coordinates the distribution of dry matter to grains from the booting stage to maturity stage, increases the number of grains per ear and thousand grain weight of winter wheat, and improves the economic yield of winter wheat [60]. The optimal proportion of substitution is less than 40%, and the nitrogen slow-release effect of excessive organic fertilizer cannot satisfy the nitrogen requirements of crop growth, thereby hindering crop yield [61]. There is no significant difference in seed cotton yield between organic fertilizer substitution treatment and single application of chemical fertilizer treatment. However, reducing the amount of chemical fertilizer and increasing the application of organic fertilizer can not only ensure crop yield, but also enhance soil fertility and promote sustainable soil utilization [62]. In this study, the combination of chemical fertilizers with organic matter and microbial fertilizers can alleviate the yield reduction effect of foxtail millet under drought stress to a certain extent, but in years with more rainfall, it may decrease the effect of chemical fertilizers and microbial fertilizers of yield. In 2017, the combination of chemical fertilizers and organic fertilizers, as well as microbial fertilizers, had the effect of improving soil quality and maintained stable yield growth in 2018 (Table 3).

### Conclusion

In this study, chemical fertilizers have the potential to increase yields directly, but considering the high efficiency and sustainability of the soil environment and fertility, organic and inorganic fertilizers can improve the internal soil environment, increase the photosynthetic and transpiration rate, chlorophyll content and nutrient utilization efficiency, and promote the accumulation and distribution of aboveground dry matter, which will further increase the yield. We showed that the NP ratio was 90:45, the above-ground biomass accumulation, transfer and transformation of the foxtail millet of spike reached maximum. Chemical fertilizer with organic manure enhanced photosynthetic rate, reduced transpiration rate, and increased above-ground dry matter accumulation, further increase crop yield. The R^2^ of nutrient organs and yield is higher when application of organic and inorganic fertilizers during the pustulation stage. Compared with nitrogen fertilizer, chemical fertilizer with organic manure or microbial manure, they significantly increased yield and WUE by 9.23%-35.17% and 8.73%-35.11%, respectively. In conclusion, organic and inorganic fertilizers have the effect of coordinating the transfer and accumulation of substances between nutrient growth and reproductive growth, improving soil environment and increasing yield.

However, our study only focuses on the two stages of growth and development of foxtail millet, we also recommend conducting long-term location experiments to conduct additional research on the combination of organic and inorganic fertilizers in different growing seasons of foxtail millet, continuously monitoring the dynamic changes in aboveground biomass, light, and parameters under the treatment of organic and inorganic fertilizers. In addition, we suggest studying the effect of the combination of organic and inorganic fertilizers on foxtail millet quality. Finally, the study on the construction of rhizosphere morphology and the interaction of microbial communities in foxtail millet treated with inorganic fertilizers combined with organic matter or microbial fertilizers is another knowledge gap.

## Supporting information

S1 File Data. (XLS)
